# Supplementary material for: An Orthogonal Electronic State View on Charge Delocalization and Transfer
Source: J Phys Chem A. 2026 Jan 20;130(4):984–91. doi: 10.1021/acs.jpca.5c07866 (PMC12862811; doi:10.1021/acs.jpca.5c07866)
Supplement: Supplementary file 1 [file jp5c07866_si_001.pdf]

Supporting information for "An Orthogonal Electronic State View on Charge Delocalization and Transfer."

Sarai Dery Folkestad* and Ida-Marie Høyvik*

Department of Chemistry, The Norwegian University of Science and Technology, Trondheim, Norway

E-mail: sarai.d.folkestad@ntnu.no; ida-marie.hoyvik@ntnu.no

Contents

1 Extended literature review **S2**

2 Properties of the charge-localized determinant basis. **S4**

3 Electron number properties of the states **S6**

4 Supporting data **S7**

4.1 The electronic coupling: comparison of basis sets and localization function . S7

4.2 The electronic coupling: comparison to results for diabatic states from the literature S9

5 A discussion on orthogonalization tails **S12**

6 **Implementation** **S12**

6.1 Localized orbitals S12

6.2 Charge-localized FCI and CISD S12

7 **Computational details** **S13**

7.1 $(H_2)_2^+$ S13

7.2 He_2^+ S13

7.3 $2H_2O$ S14

7.4 Benzene-chlorine S14

1 Extended literature review

Information on whether there is sufficient electronic coupling between two molecules to induce integer or partial electron transfer, can be found in the Hamiltonian for the interacting composite system. These electronic couplings have significance for several important fields, such as electron transfer theory[1–5] and bilinear coupling elements in theoretical models of molecular electronics.[6–8] Further, it allows for parametrizing multiscale models with electronically open boundary conditions by using wave function models with electron-number fluctuations.[9–12] In particular, the use of electronic coupling elements to predict electron transfer rates through e.g., Marcus theory[1] has resulted in a large interest in computing such couplings for many decades.[4,13–37] The electronic coupling is the matrix element of the Hamiltonian sandwiched between diabatic states representing the initial and final electronic states[38,39] of the electron transfer process. Diabatic states are formally states in which the nuclear derivative coupling is zero (or small),[40–42] but Mead and Truhlar have found that these do not in general exist.[43] Hence, the term diabatic is more commonly used for states which fulfill some chosen criteria, for example by designing diabatic states with desirable characteristics[21–23,44–46] or invoking some physical observable such as the dipole operator.[16,17] In contrast, states which diagonalize the electronic Hamiltonian are called adiabatic states,[40]

and (approximate) solutions to the electronic Schrödinger equation are therefore usually referred to as adiabatic states.

In what Atchity and Ruedeberg refer to as the electronic-structure context,[21,22] as opposed to the nonadiabatic dynamics context, diabatic states are states *"whose electronic structures maintain their essential characteristics"* in certain regions of coordinate space.[21] Atchity and Rudenberg deduced an orthogonal transformation from adiabatic to diabatic states by analysing the adiabatic configuration interaction (CI) coefficients. The diabatic states were defined to be those for which the dominant configuration were unchanged for the given coordinate space. A conceptually different approach is the Boys localization of states by Subotnik et al.[27] They use a localization criteria usually defined for orbitals[47,48] to rotate adiabatic states into appropriate diabatic states. It can be viewed as a generalized Mulliken-Hush[16] generalized to multiple charge centers, as shown in Ref. 27. Further, a wide range of DFT variants have been developed to obtain diabatic states and/or electronic coupling elements[19,20,25,29,34,49–55] In the context of frozen density embedding (FDE) DFT, Pavanello and Neugebauer refer to the FDE states as charge-localized rather than diabatic.[19] However, they note that the FDE states are configurationally uniform across an electron-transfer reaction coordinate and hence can be considered (quasi-)diabatic. A recent tutorial by Neugebauer and collaborators give a good description of how to construct diabatic states within fragment based quantum chemistry.[56] Further, various valence bond approaches are also used in connection with computing diabatic states.[25,31,33,57]

Requiring determinants to reflect electron distributions offers a clear and simple interpretation of adiabatic states as the "resonance hybrids"[58] between different electron distributions. This is analogous to resonances between Lewis structures in covalent bonding, except that here we are not interested in details within a specific molecule but rather between molecules or between different regions in a molecule. Hence, integer electron transfer as well as partial ionic characters can be explained with equal terminology and ease as is used to explain covalent bonding and partial ionic characters of covalent bonds, and it can

be extracted directly from the electronic wave function. This is useful in terms of discussing bonding situations, such as hydrogen bonding. The role of partial ionic character (resonances between neutral and ionic charge distributions) in hydrogen bonding was early discussed in the literature.[59–63] However, as pointed out by Weinhold and Klein[64] as late as in 2012, most current textbooks describe hydrogen bonding with wording which only reflect the classical electrostatic picture (see discussion in Ref. 64). Although there seem to be little controversy regarding that there is charge-transfer in hydrogen bonds, the amount is under debate.[65,66]

The charge-localized CI states introduced in the letter, comply with Atchity and Ruedenbergs definition of configurational uniformity in certain regions of coordinate space.[21] However, we refrain from using the term "diabatic" and rather choose "charge-localized" (as also used for some of the DFT variants discussed above). The reason for this is twofold. First, the charge-localized states are generated by staying within a specific electron distribution among interacting subsystems, and the term "charge-localized" is therefore correctly descriptive. Second, the charge-localized states is the result of diagonalizing an approximate electronic Hamiltonian (for each nuclear configuration), where matrix elements connecting different electron distributions are neglected. As such, it fits with Smith's definition[40] of an (approximate) adiabatic state as defined by the requirement that the electronic Hamiltonian is diagonal for each nuclear configuration. Hence, we prefer to think of the concept "diabatic" in connection with small nuclear derivative coupling elements[40–42] important for nonadiabatic dynamics. However, this is not within the scope of the presented framework, in which charge-transfer and related concepts are the objective.

2 Properties of the charge-localized determinant basis.

In the CI and charge-localized CI calculations, we use a charge-localized determinant basis,

$$\left|I^{\lambda}\right\rangle : \quad \text{determinant } I \text{ with electron distribution } (N_A+\lambda, N_B-\lambda), \tag{1}$$

$\lambda = 0, \pm 1, \pm 2, \cdots$. We have chosen a convention where subsystem A has received λ electrons from B, relative to the reference. I.e, if $\lambda = 1$, subsystem A has $N_A + 1$ electrons while subsystem B has $N_B - 1$ electrons. Similarly, $\lambda = -1$ gives the opposite case. We note that $\lambda = 0$ represents the reference electron distribution with N_A electrons on A and N_B electrons on B. Furthermore, we note that all determinants are built from a common set of orthonormal orbitals ($\{\phi_p\} \cup \{\phi_{\bar{p}}\}$) and hence the determinants make up a set of orthonormal N-electron determinants,

$$\left\langle I^\lambda \middle| J^\tau \right\rangle = \delta_{IJ}\delta_{\lambda\tau}. \tag{2}$$

In the local spin-orbital basis, the number operator for the composite system naturally separates into a number operator for subsystem A and a number operator for subsystem B,

$$\hat{n} = \sum_p a_p^\dagger a_p + \sum_{\bar{p}} a_{\bar{p}}^\dagger a_{\bar{p}} = \hat{n}_A + \hat{n}_B. \tag{3}$$

The charge-localized determinants $\left| I^\lambda \right\rangle$ are eigenstates of the number operators $\hat{n}_A$ and $\hat{n}_B$, where the eigenvalues depend on λ,

$$\begin{aligned} \hat{n}_A \left| I^\lambda \right\rangle &= (N_A + \lambda) \left| I^\lambda \right\rangle \\ \hat{n}_B \left| I^\lambda \right\rangle &= (N_B - \lambda) \left| I^\lambda \right\rangle. \end{aligned} \tag{4}$$

Furthermore, all determinants $\left| I^\lambda \right\rangle$ are eigenstates with the same eigenvalue of the total number operator,

$$(\hat{n}_A + \hat{n}_B) \left| I^\lambda \right\rangle = N \left| I^\lambda \right\rangle, \tag{5}$$

where N is the total number of electrons in the composite system.

3 Electron number properties of the states

The CI wave function for state k is,

$$|\Psi_k\rangle = \sum_{\lambda}\sum_{I^\lambda} C_k^{I^\lambda} \left|I^\lambda\right\rangle \tag{6}$$

In the charge-localized determinant basis, we have direct access to computing charge distributions on either of the interacting molecules via expectation values. For example, the average number of electrons on subsystem A for the ground state ($k = 0$), is given by

$$\langle \hat{n}_A \rangle_0 = \langle \Psi_0 | \, \hat{n}_A \, |\Psi_0\rangle = \sum_{\lambda}\sum_{I^\lambda} |C_0^{I^\lambda}|^2 (N_A + \lambda). \tag{7}$$

This follows from eq. (4), and a similar relation hold for subsystem B. However, since $|\Psi_k\rangle$ is not necessarily an eigenfunction of $\hat{n}_A$ and $\hat{n}_B$, separately, there will be a non-zero variance in the electron number computed for A and B. E.g., for state $|\Psi_k\rangle$ we have in general,

$$\begin{aligned} \langle \hat{n}_A^2 \rangle_k - \langle \hat{n}_A \rangle_k^2 &\neq 0 \\ \langle \hat{n}_B^2 \rangle_k - \langle \hat{n}_B \rangle_k^2 &\neq 0. \end{aligned} \tag{8}$$

In contrast, there is no spread in the total number of electrons,

$$\langle \hat{n}^2 \rangle_k - \langle \hat{n} \rangle_k^2 = 0, \tag{9}$$

since all determinants have N electrons (see eq. (5)).

4 Supporting data

4.1 The electronic coupling: comparison of basis sets and localization function

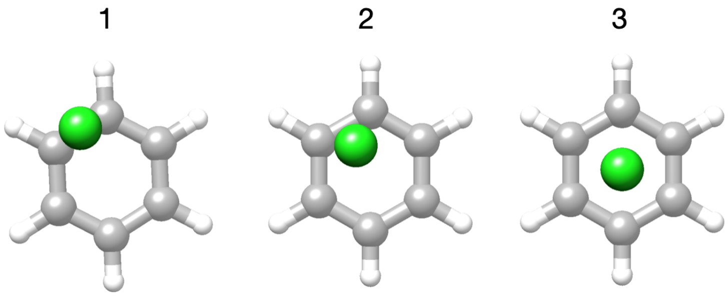

Figure S1: Visualization[67] of the three geometries of benzene and chlorine atom (geometries named 1-3) for which coupling elements are computed. The chlorine atom is placed 3 Å above the benzene ring plane.

To evaluate the dependence of the charge-localized CISD approach on basis set and localization function, we consider a neutral system of a benzene molecule interacting with a chlorine atom which lies in the plane 3 Å above the benzene molecular plane. We consider three different geometries, visualized in Figure S1. This benzene-chlorine system has been used by extensively to benchmark approximations for the electronic coupling elements.[16,17,50,68]

In this work, localized orbitals are obtained from a closed-shell Hartree–Fock calculation of the system with an additional electron. However, for benzene-chlorine, restricted open-shell Hartree–Fock orbitals can be used, since the unpaired electron is located on the chlorine atom in the reference determinant. In Table S1, we present the coupling element between the charge-localized ground state where both benzene and chlorine are neutral ($\lambda = 0$), and the charge-localized ground state where an electron has been transferred to the chlorine ($\lambda = -1$). That is, we consider $|\tilde{H}_{00}^{0-1}|$. Table S2 shows the expected number of electrons for benzene in the ground and excited adiabatic states. The calculations have been performed with the Foster-Boys[69] and Edmiston-Ruedenberg[48] localization functions, using the cc-

pVDZ, 6-31+g*, and aug-cc-pVDZ basis sets.

Table S1: Coupling elements $|\tilde{H}_{00}^{0-1}|$ for Benzene-Cl for geometries 1-3 (displayed in Figure S1) computed using charge-localized CISD with Foster-Boys or Edmiston-Ruedenberg localization. All results are in eV

	Foster-Boys			Edmiston-Ruedenberg		
	1	2	3	1	2	3
cc-pVDZ	0.51	0.38	0.00	0.51	0.38	0.00
6-31+g*	0.55	0.41	0.00	0.52	0.39	0.01
aug-cc-pVDZ	0.56	0.45	0.02	0.52	0.41	0.00

Table S2: Expected number of electrons for benzene, $\langle n_{\text{benzene}} \rangle$, in benzene-chlorine for geometries 1-3 (displayed in Figure S1) computed for ground and excited states using CISD in the charge-localized determinant basis and eq. (7) with either Foster-Boys Edmiston-Ruedenberg localization

	Foster-Boys					
	Ground state			Excited state		
Geometry	1	2	3	1	2	3
cc-pVDZ	42.00	42.00	42.00	41.94	41.93	41.89
6-31+g*	42.00	42.00	42.00	41.89	41.85	41.96
aug-cc-pVDZ	42.00	42.00	42.00	41.87	41.83	41.95
	Edmiston-Ruedenberg					
	Ground state			Excited state		
Geometry	1	2	3	1	2	3
cc-pVDZ	42.00	42.00	42.00	41.93	41.91	41.89
6-31+g*	42.00	42.00	42.00	41.90	41.86	41.96
aug-cc-pVDZ	42.00	42.00	42.00	41.88	41.84	41.95

Whereas the coupling elements (see Table S1) are qualitatively similar irrespective of basis set, the change in $\langle n_{\text{benzene}} \rangle$ as the chlorine moves towards the center of the benzene ring (see Table S2) is characteristically different when augmentation functions are included in the basis set. We note that the localized Hartree-Fock orbitals are not symmetry-adapted, and that the resulting charge-localized states may contain errors due to symmetry-breaking. These errors increase with the addition of augmentation functions to the basis set. On the other hand, such functions are likely important to correctly capture the partial anionic

character of the electron acceptor. For systems with high degree of symmetry, localizing the Hartree-Fock orbitals in symmetry blocks will ensure a symmetry-adapted treatment.

The two different localization functions qualitatively yield the same picture for both $\langle n_{\text{benzene}} \rangle$ and $|\tilde{H}_{00}^{0-1}|$, despite the differences in the type of localized orbitals resulting from the two approaches. This is because, while individual orbitals generated by the two localization schemes are different, only the total electronic density on the subsystems matter for the CI framework presented here.

4.2 The electronic coupling: comparison to results for diabatic states from the literature

In Table S3, we have reproduced the coupling elements of the benzene-chlorine system (see Figure S1) obtained with with generalized Mulliken-Hush and valence-bond block-diagonalization from Refs. 16 and 68. In both these approaches, the coupling elements are characterized according to the p-orbitals of the chlorine atom, e.g., it is the coupling with respect to transfer of electrons from benzene to chlorine. We will define the charge-localized states such that this corresponds to $\lambda = -1$. To compare the charge-localized CISD coupling elements (a state picture) to the approaches above (characterized by orbitals), we plot density differences of charge-localized states to see the orbital density character of the change. The density difference are defined through:

$$[\boldsymbol{\Delta}\tilde{\boldsymbol{\rho}}_{mn}^{-10}]_{PQ} = [\tilde{\boldsymbol{\rho}}_{m}^{-1}]_{PQ} - [\tilde{\boldsymbol{\rho}}_{n}^{0}]_{PQ} = \left\langle \tilde{\Psi}_{m}^{-1} \middle| a_{P}^{\dagger} a_{Q} \middle| \tilde{\Psi}_{m}^{-1} \right\rangle - \left\langle \tilde{\Psi}_{n}^{0} \middle| a_{P}^{\dagger} a_{Q} \middle| \tilde{\Psi}_{n}^{0} \right\rangle . \qquad (10)$$

The density differences between the charge localized states corresponding to the first large coupling element obtained with the cc-pVDZ basis are plotted in Figure S2; blue indicates electron density addition and red indicates electron density removal. We place benzene in the xy-plane. The xz-plane is the mirror plane for geometries 1 and 2 (C_s point-group), and the z-axis is the C_6-axis for geometry 3 (C_{6v} point-group). For geometry 1, we can

see that the appropriate comparison for $|H_{00}^{0-1}| = 0.51$ are the $|H_{p_x}|$ and $|H_{p_z}|$ elements of generalized Mulliken-Hush and valence-bond block diagonalization. The charge localized CISD coupling element is consistent in magnitude ($|H_{p_x}| < |H_{00}^{0-1}| < |H_{p_z}|$). For geometry 2, we can see that the appropriate comparison for $|H_{00}^{0-1}| = 0.38$ is the $|H_{p_z}|$ coupling element of generalized Mulliken-Hush and valence-bond block diagonalization. Again, the magnitude of the coupling elements obtained with the three approaches are consistent. Finally, for geometry 3, we consider the coupling elements $|H_{10}^{0-1}| = |H_{21}^{0-1}| = 0.54$. The corresponding charge localized states are degenerate pairs spanning the E_1 irreducible representation. The density differences are plotted together in Figure S2c. From Table S3, we can see that the coupling elements equals those reported with the valence-bond block-diagonalization approach.

Table S3: Coupling elements reported by the Generalized Mulliken-Hush[16,17](GMH) and the valence-bond block diagonalization (VBBD) approaches. The VBBD results are obtained with a 6-31+g* basis set and the GMH results with a VDZ bases set. All results are given in eV

	GMH		VBBD		
	1	2	1	2	3
$\lvert H_{p_x}\rvert$	0.36	0.40	0.27	0.41	0.54†
$\lvert H_{p_y}\rvert$	0.40	0.50	0.41	0.50	0.54†
$\lvert H_{p_z}\rvert$	0.66	0.41	0.72	0.49	0.00

† Two-state approach

Coupling elements for the benzene-chlorine system has also been calculated using the constrained DFT approach in Ref. 50 by Wu and Van Voorhis, they calculate coupling elements that are significantly larger in magnitude using Becke weight populations; the use of Löwdin populations results in coupling elements that are heavily basis set dependent.

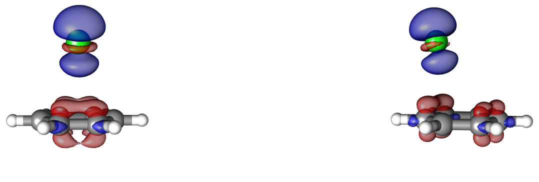

(a) Geometry 1: $\boldsymbol{\Delta}\tilde{\boldsymbol{\rho}}_{00}^{-10} = \tilde{\boldsymbol{\rho}}_0^{-1} - \tilde{\boldsymbol{\rho}}_0^0$ plotted from two angles. Red indicates electron density removal and blue indicates electron energy addition. The corresponding coupling element is $|H_{00}^{0-1}| = 0.509$.

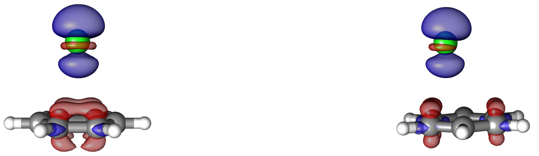

(b) Geometry 2: $\boldsymbol{\Delta}\tilde{\boldsymbol{\rho}}_{00}^{-10} = \tilde{\boldsymbol{\rho}}_0^{-1} - \tilde{\boldsymbol{\rho}}_0^0$, plotted from two angles. Red indicates electron density removal and blue indicates electron energy addition. The corresponding coupling element is $|H_{00}^{0-1}| = 0.375$.

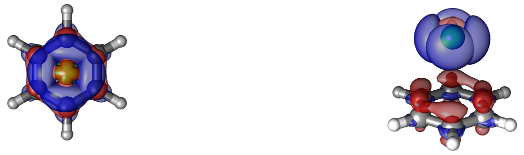

(c) Geometry 3: $\boldsymbol{\Delta}\tilde{\boldsymbol{\rho}}_{01}^{-10} = \tilde{\boldsymbol{\rho}}_0^{-1} - \tilde{\boldsymbol{\rho}}_1^0$ and $\boldsymbol{\Delta}\tilde{\boldsymbol{\rho}}_{12}^{-10} = \tilde{\boldsymbol{\rho}}_1^{-1} - \tilde{\boldsymbol{\rho}}_2^0$ plotted together, from two angles. Red indicates electron density removal and blue indicates electron energy addition. The corresponding coupling elements are $|H_{10}^{0-1}| =$ and $|H_{21}^{0-1}| = 0.54$

Figure S2: Density difference (in the cc-pVDZ basis) (at an isovalue of 0.01 a.u.) between charge localized states

5 A discussion on orthogonalization tails

From the results above, it is clear that the CISD wave function dictates that a small amount of electronic charge (on the millielectron scale) is transferred from one water molecule to the other upon interaction, and that the presence of the ionic contributions are important for the wave function. One may argue that results are blurred by the fact that orbitals on one molecule have small components (orthogonalization tails) on the other molecule. In other words, that there is in fact no charge-transfer and that it is a artifact of the orthogonalization tails. If this was the case, one would also see that anionic-cationic distributions (measured by P_0^1) would exhibit similiar numerics to the cationic-anionic distributions (measured by P_0^{-1}). However, this is not the case.

6 Implementation

6.1 Localized orbitals

The localization of the orbitals is carried out using the trust-region optimization algorithm for orbital localization functions[70] as implemented in the $e^{\mathcal{T}}$ program,[71] using either the Edmiston-Ruedenberg or the Foster-Boys localization functions.[48] The implementation is described in Ref. 72. The localized orbitals are assigned to the subsystems and enables the construction of charge-localized determinants. These determinants are used in the subsequent CI and charge-localized CI calculations.

6.2 Charge-localized FCI and CISD

The FCI implementation for charge-localized states is based on a determinant string implementation of FCI, as detailed in Ref. 73. The CISD implementation is in the spin-orbital basis. The CI states are determined by diagonalizing eq. (5). The charge-localized states are

identified by diagonalizing Hamiltonian matrix in eq. (9). In practice, this is achieved using a Davidson algorithm for the full Hamiltonian (eq. (5)) with projections that remove all components of the trial and transformed vectors that does not correspond to the given λ. Once this procedure has been performed separately for the relevant λ-values, we may compute the coupling matrix between the states corresponding to two different electron distributions.

7 Computational details

Here, we specify the computational details for the calculations presented in the manuscript and the *Supporting Information*. For all correlated calculations in the $e^{\mathcal{T}}$ program uses Cholesky decomposition of the electron repulsion integrals. A decomposition threshold of 10^{-11} is used throughout, and effectively eliminates any approximation due to such factorization.

7.1 $(\mathbf{H}_2)_2^+$

The FCI calculations on $(H_2)_2^+$/cc-pVDZ are performed with localized Hartree–Fock orbitals for the neutral system. The threshold for the Hartree–Fock gradient is 10^{-11} (maximum norm) and the orbital localization threshold is 10^{-7} (l_2-norm). In these calculations we have used the Edmiston-Ruedenberg[48] localization function. The FCI and charge-localized FCI calculations have been converged to a residual norm (l_2-norm) of $< 10^{-7}$.

7.2 $\mathbf{He}_2^+$

The FCI calculations on $(H_2)_2^+$/6-31g* are performed with localized Hartree–Fock orbitals for the neutral system. The threshold for the Hartree–Fock gradient is 10^{-11} (maximum norm) and the orbital localization threshold is 10^{-7} (l_2-norm). In these calculations we have used the Edmiston-Ruedenberg[48] localization function. The FCI and charge-localized FCI calculations have been converged to a residual norm (l_2-norm) of $< 10^{-7}$.

7.3 $2H_2O$

The CISD calculations on $2H_2O$/aug-cc-pVDZ are performed with localized Hartree–Fock orbitals. The threshold for the Hartree–Fock gradient is 10^{-11} (maximum norm) and the orbital localization threshold is 10^{-5} (l_2-norm). In these calculations we have used the Foster-Boys[69] localization function. The CISD and charge-localized CISD calculations have been converged to a residual norm (l_2-norm) of $< 10^{-7}$.

7.4 Benzene-chlorine

The CISD calculations on the benzene-chlorine are performed with localized Hartree–Fock orbitals at the closed-shell anionic system. The threshold for the Hartree–Fock gradient is 10^{-11} (maximum norm) except for the aug-cc-pVDZ where the Hartree-Fock orbitals were converged to 10^{-8}. The orbital localization threshold is 10^{-5} (l_2-norm). In these calculations we have used the Foster-Boys[69] localization function. The CISD and charge-localized CISD calculations have been converged to a residual norm (l_2-norm) of $< 10^{-4}$.

References

(1) Marcus, R. A. On the Theory of Oxidation-Reduction Reactions Involving Electron Transfer. I. *The Journal of Chemical Physics* **1956**, *24*, 966–978.

(2) Barbara, P. F.; Meyer, T. J.; Ratner, M. A. Contemporary Issues in Electron Transfer Research. *J. Phys. Chem.* **1996**, *100*, 13148–13168, Publisher: American Chemical Society.

(3) Van Voorhis, T.; Kowalczyk, T.; Kaduk, B.; Wang, L.-P.; Cheng, C.-L.; Wu, Q. The Diabatic Picture of Electron Transfer, Reaction Barriers, and Molecular Dynamics. *Annual Review of Physical Chemistry* **2010**, *61*, 149–170.

(4) Hsu, C.-P. The Electronic Couplings in Electron Transfer and Excitation Energy Transfer. *Accounts of Chemical Research* **2009**, *42*, 509–518.

(5) Futera, Z.; Blumberger, J. Electronic Couplings for Charge Transfer across Molecule/Metal and Molecule/Semiconductor Interfaces: Performance of the Projector Operator-Based Diabatization Approach. *The Journal of Physical Chemistry C* **2017**, *121*, 19677–19689.

(6) Nitzan, A. Electron transmission through molecules and molecular interfaces. *Annual Review of Physical Chemistry* **2001**, *52*, 681–750.

(7) Nitzan, A. A Relationship between Electron-Transfer Rates and Molecular Conduction. *The Journal of Physical Chemistry A* **2001**, *105*, 2677–2679.

(8) Naaman, R.; Waldeck, D. H.; Fransson, J. New Perspective on Electron Transfer through Molecules. *The Journal of Physical Chemistry Letters* **2022**, *13*, 11753–11759.

(9) Matveeva, R.; Folkestad, S. D.; Høyvik, I.-M. Particle-Breaking Hartree–Fock Theory for Open Molecular Systems. *The Journal of Physical Chemistry A* **2023**, *127*, 1329–1341.

(10) Paul née Matveeva, R.; Folkestad, S. D.; Sannes, B. S.; Høyvik, I.-M. Particle-Breaking Unrestricted Hartree–Fock Theory for Open Molecular Systems. *The Journal of Physical Chemistry A* **2024**, *128*, 1533–1542.

(11) Pedersen, J.; Sannes, B. S.; Paul née Matveeva, R.; Coriani, S.; Høyvik, I.-M. Time-Dependent Particle-Breaking Hartree–Fock Model for Electronically Open Molecules. *The Journal of Physical Chemistry A* **2025**, *129*, 4288–4300.

(12) Sannes, B. S.; Pedersen, J.; Høyvik, I.-M. Fractional charging of electronically open molecules: An explicit projection operator approach. *The Journal of Chemical Physics* **2025**, *162*, 094108.

(13) Marcus, R. A.; Sutin, N. Electron transfers in chemistry and biology. *Biochimica et Biophysica Acta (BBA) - Reviews on Bioenergetics* **1985**, *811*, 265–322.

(14) Newton, M. D. Quantum chemical probes of electron-transfer kinetics: the nature of donor-acceptor interactions. *Chemical Reviews* **1991**, *91*, 767–792.

(15) Braga, M.; Larsson, S. Electronic factor for electron transfer through cyclohexane-type spacers. *The Journal of Physical Chemistry* **1993**, *97*, 8929–8936.

(16) Cave, R. J.; Newton, M. D. Generalization of the Mulliken-Hush treatment for the calculation of electron transfer matrix elements. *Chemical Physics Letters* **1996**, *249*, 15–19.

(17) Cave, R. J.; Newton, M. D. Calculation of electronic coupling matrix elements for ground and excited state electron transfer reactions: Comparison of the generalized Mulliken–Hush and block diagonalization methods. *The Journal of Chemical Physics* **1997**, *106*, 9213–9226.

(18) Hsu, C.-P.; Marcus, R. A. A sequential formula for electronic coupling in long range bridge-assisted electron transfer: Formulation of theory and application to alkanethiol monolayers. *The Journal of Chemical Physics* **1997**, *106*, 584–598.

(19) Pavanello, M.; Neugebauer, J. Modelling charge transfer reactions with the frozen density embedding formalism. *The Journal of Chemical Physics* **2011**, *135*, 234103.

(20) Migliore, A. Full-electron calculation of effective electronic couplings and excitation energies of charge transfer states: Application to hole transfer in DNA -stacks. *The Journal of Chemical Physics* **2009**, *131*, 114113.

(21) Atchity, G. J.; Ruedenberg, K. Determination of diabatic states through enforcement of configurational uniformity. *Theoretical Chemistry Accounts* **1997**, *97*, 47–58.

(22) Ruedenberg, K.; Atchity, G. J. A quantum chemical determination of diabatic states. *The Journal of Chemical Physics* **1993**, *99*, 3799–3803.

(23) Nakamura, H.; Truhlar, D. G. The direct calculation of diabatic states based on configurational uniformity. *The Journal of Chemical Physics* **2001**, *115*, 10353–10372.

(24) Nakamura, H.; Truhlar, D. G. Extension of the fourfold way for calculation of global diabatic potential energy surfaces of complex, multiarrangement, non-Born–Oppenheimer systems: Application to HNCO(S,S1). *The Journal of Chemical Physics* **2003**, *118*, 6816–6829.

(25) Cembran, A.; Song, L.; Mo, Y.; Gao, J. Block-Localized Density Functional Theory (BLDFT), Diabatic Coupling, and Their Use in Valence Bond Theory for Representing Reactive Potential Energy Surfaces. *Journal of Chemical Theory and Computation* **2009**, *5*, 2702–2716.

(26) Futera, Z.; Blumberger, J. Electronic Couplings for Charge Transfer across Molecule/Metal and Molecule/Semiconductor Interfaces: Performance of the Projector Operator-Based Diabatization Approach. *J. Phys. Chem. C* **2017**, *121*, 19677–19689.

(27) Subotnik, J. E.; Yeganeh, S.; Cave, R. J.; Ratner, M. A. Constructing diabatic states from adiabatic states: Extending generalized Mulliken–Hush to multiple charge centers with Boys localization. *The Journal of Chemical Physics* **2008**, *129*, 244101.

(28) Kondov, I.; Čížek, M.; Benesch, C.; Wang, H.; Thoss, M. Quantum Dynamics of Photoinduced Electron-Transfer Reactions in DyeSemiconductor Systems: First-Principles Description and Application to Coumarin 343-TiO2. *The Journal of Physical Chemistry C* **2007**, *111*, 11970–11981.

(29) Pavanello, M.; Van Voorhis, T.; Visscher, L.; Neugebauer, J. An accurate and linear-scaling method for calculating charge-transfer excitation energies and diabatic couplings. *The Journal of Chemical Physics* **2013**, *138*, 054101.

(30) Gray, H. B.; Winkler, J. R. Long-range electron transfer. *Proceedings of the National Academy of Sciences* **2005**, *102*, 3534–3539, Publisher: Proceedings of the National Academy of Sciences.

(31) Lin, X.; Liu, X.; Ying, F.; Chen, Z.; Wu, W. Explicit construction of diabatic state and its application to the direct evaluation of electronic coupling. *The Journal of Chemical Physics* **2018**, *149*, 044112.

(32) Pourtois, G.; Beljonne, D.; Cornil, J.; Ratner, M. A.; Brédas, J. L. Photoinduced Electron-Transfer Processes along Molecular Wires Based on Phenylenevinylene Oligomers: A Quantum-Chemical Insight. *J. Am. Chem. Soc.* **2002**, *124*, 4436–4447.

(33) Song, L.; Gao, J. On the Construction of Diabatic and Adiabatic Potential Energy Surfaces Based on Ab Initio Valence Bond Theory. *J. Phys. Chem. A* **2008**, *112*, 12925–12935.

(34) Grofe, A.; Qu, Z.; Truhlar, D. G.; Li, H.; Gao, J. Diabatic-At-Construction Method for Diabatic and Adiabatic Ground and Excited States Based on Multistate Density Functional Theory. *Journal of Chemical Theory and Computation* **2017**, *13*, 1176–1187.

(35) Biancardi, A.; Martin, S. C.; Liss, C.; Caricato, M. Electronic Coupling for Donor-Bridge-Acceptor Systems with a Bridge-Overlap Approach. *Journal of Chemical Theory and Computation* **2017**, *13*, 4154–4161.

(36) Storm, F. E.; Rasmussen, M. H.; Mikkelsen, K. V.; Hansen, T. Computational construction of the electronic Hamiltonian for photoinduced electron transfer and Redfield propagation. *Physical Chemistry Chemical Physics* **2019**, *21*, 17366–17377.

(37) Illésová, S.; Beseda, M.; Yalouz, S.; Lasorne, B.; Senjean, B. Transformation-Free Generation of a Quasi-Diabatic Representation from the State-Average Orbital-Optimized Variational Quantum Eigensolver. *Journal of Chemical Theory and Computation* **2025**, *21*, 5457–5480.

(38) Mikkelsen, K. V.; Dalgaard, E.; Swanstroem, P. Electron-transfer reactions in solution: an ab initio approach. *The Journal of Physical Chemistry* **1987**, *91*, 3081–3092.

(39) Subotnik, J. E.; Cave, R. J.; Steele, R. P.; Shenvi, N. The initial and final states of electron and energy transfer processes: Diabatization as motivated by system-solvent interactions. *The Journal of Chemical Physics* **2009**, *130*, 234102.

(40) Smith, F. T. Diabatic and Adiabatic Representations for Atomic Collision Problems. *Physical Review* **1969**, *179*, 111–123.

(41) Baer, M. Adiabatic and diabatic representations for atom-molecule collisions: Treatment of the collinear arrangement. *Chemical Physics Letters* **1975**, *35*, 112–118.

(42) O'Malley, T. F. In *Advances in Atomic and Molecular Physics*; Bates, D. R., Esterman, I., Eds.; Academic Press, 1971; Vol. 7; pp 223–249.

(43) Mead, C. A.; Truhlar, D. G. Conditions for the definition of a strictly diabatic electronic basis for molecular systems. *The Journal of Chemical Physics* **1982**, *77*, 6090–6098.

(44) Accomasso, D.; Persico, M.; Granucci, G. Diabatization by Localization in the Framework of Configuration Interaction Based on Floating Occupation Molecular Orbitals (FOMO-CI). *ChemPhotoChem* **2019**, *3*, 933–944.

(45) Pacher, T.; Cederbaum, L. S.; Köppel, H. Approximately diabatic states from block diagonalization of the electronic Hamiltonian. *The Journal of Chemical Physics* **1988**, *89*, 7367–7381.

(46) Hiberty, P. C.; Shaik, S. Breathing-orbital valence bond method –a modern valence bond method that includes dynamic correlation. *Theoretical Chemistry Accounts* **2002**, *108*, 255–272.

(47) Foster, J. M.; Boys, S. F. Canonical Configurational Interaction Procedure. *Rev. Mod. Phys.* **1960**, *32*, 300–302.

(48) Edmiston, C.; Ruedenberg, K. Localized Atomic and Molecular Orbitals. *Rev. Mod. Phys.* **1963**, *35*, 457–464.

(49) Wu, Q.; Van Voorhis, T. Direct optimization method to study constrained systems within density-functional theory. *Phys. Rev. A* **2005**, *72*, 024502.

(50) Wu, Q.; Van Voorhis, T. Constrained Density Functional Theory and Its Application in Long-Range Electron Transfer. *J. Chem. Theory Comput.* **2006**, *2*, 765–774.

(51) Hong, G.; Rosta, E.; Warshel, A. Using the Constrained DFT Approach in Generating Diabatic Surfaces and Off Diagonal Empirical Valence Bond Terms for Modeling Reactions in Condensed Phases. *The Journal of Physical Chemistry B* **2006**, *110*, 19570–19574.

(52) Xiang, Y.; Warshel, A. Quantifying Free Energy Profiles of Proton Transfer Reactions in Solution and Proteins by Using a Diabatic FDFT Mapping. *The Journal of Physical Chemistry B* **2008**, *112*, 1007–1015.

(53) Oberhofer, H.; Blumberger, J. Electronic coupling matrix elements from charge constrained density functional theory calculations using a plane wave basis set. *The Journal of Chemical Physics* **2010**, *133*, 244105.

(54) Ramos, P.; Papadakis, M.; Pavanello, M. Performance of Frozen Density Embedding for Modeling Hole Transfer Reactions. *The Journal of Physical Chemistry B* **2015**, *119*, 7541–7557.

(55) Ren, H.; Provorse, M. R.; Bao, P.; Qu, Z.; Gao, J. Multistate Density Functional Theory for Effective Diabatic Electronic Coupling. *The Journal of Physical Chemistry Letters* **2016**, *7*, 2286–2293.

(56) Rikus, A.; Käfer, S.; Lampe, L.; Mück-Lichtenfeld, C.; Tölle, J.; Neugebauer, J. How to

Construct Diabatic States for Energy and Charge Transfer with Subsystem Quantum ChemistryA Tutorial. *The Journal of Physical Chemistry A* **2025**, *129*, 7238–7250.

(57) Ren, M.; Liu, X.; Zhang, L.; Lin, X.; Wu, W.; Chen, Z. Compact and accurate ab initio valence bond wave functions for electron transfer: The classic but challenging covalent-ionic interaction in LiF. *The Journal of Chemical Physics* **2022**, *157*, 084106.

(58) Pauling, L. *The nature of the chemical bond*, 3rd ed.; Cornell University Press, 1960.

(59) Coulson, C. A. *Valence*, 2nd ed.; Oxxford University Press, 1952.

(60) Bratož, S. In *Advances in Quantum Chemistry*; Löwdin, P.-O., Ed.; Academic Press, 1967; Vol. 3; pp 209–237.

(61) Pimentel, G. C.; McClellan, A. L. Hydrogen Bonding. *Annual Review of Physical Chemistry* **1971**, *22*, 347–385.

(62) Ratajczak, H.; Orville-Thomas, W. J. Charge-transfer properties of hydrogen bonds. III. Charge-transfer theory and the relation between the energy and the enhancement of dipole moment of hydrogen-bonded complexes. *The Journal of Chemical Physics* **1973**, *58*, 911–919.

(63) Reed, A. E.; Curtiss, L. A.; Weinhold, F. Intermolecular interactions from a natural bond orbital, donor-acceptor viewpoint. *Chemical Reviews* **1988**, *88*, 899–926.

(64) Weinhold, F.; Klein, R. A. What is a hydrogen bond? Mutually consistent theoretical and experimental criteria for characterizing H-bonding interactions. *Molecular Physics* **2012**, *110*, 565–579.

(65) Stone, A. J. Natural Bond Orbitals and the Nature of the Hydrogen Bond. *The Journal of Physical Chemistry A* **2017**, *121*, 1531–1534.

(66) Weinhold, F.; Glendening, E. D. Comment on "Natural Bond Orbitals and the Nature of the Hydrogen Bond". *The Journal of Physical Chemistry A* **2018**, *122*, 724–732.

(67) Pettersen, E. F.; Goddard, T. D.; Huang, C. C.; Couch, G. S.; Greenblatt, D. M.; Meng, E. C.; Ferrin, T. E. UCSF Chimera–a visualization system for exploratory research and analysis. *Journal of Computational Chemistry* **2004**, *25*, 1605–1612.

(68) Wu, Q.; Van Voorhis, T. Extracting electron transfer coupling elements from constrained density functional theory. *The Journal of Chemical Physics* **2006**, *125*, 164105.

(69) Boys, S. F. Construction of Some Molecular Orbitals to Be Approximately Invariant for Changes from One Molecule to Another. *Rev. Mod. Phys.* **1960**, *32*, 296–299.

(70) Høyvik, I.-M.; Jansik, B.; Jørgensen, P. Trust Region Minimization of Orbital Localization Functions. *Journal of Chemical Theory and Computation* **2012**, *8*, 3137–3146.

(71) Folkestad, S. D. et al. eT 1.0: An open source electronic structure program with emphasis on coupled cluster and multilevel methods. *The Journal of Chemical Physics* **2020**, *152*, 184103.

(72) Folkestad, S. D.; Matveeva, R.; Høyvik, I.-M.; Koch, H. Implementation of Occupied and Virtual Edmiston–Ruedenberg Orbitals Using Cholesky Decomposed Integrals. *Journal of Chemical Theory and Computation* **2022**, *18*, 4733–4744.

(73) Helgaker, T.; Jørgensen, P.; Olsen, J. *Molecular electronic-structure theory*; John Wiley & Sons, 2013.
